# Multi-factor regulatory network and different clusters in hypertrophic obstructive cardiomyopathy

**DOI:** 10.1186/s12920-021-01036-4

**Published:** 2021-08-06

**Authors:** Xianyu Qin, Lei Huang, Sicheng Chen, Shaoxian Chen, Pengju Wen, Yueheng Wu, Jian Zhuang

**Affiliations:** 1 Department of Thoracic Surgery, Thoracic Cancer Center, The Sixth Affiliated Hospital of Sun Yat-Sen University, Guangzhou, China; 2 Department of Cardiovascular Surgery, Guangdong Cardiovascular Institute, Guangdong Provincial Key Laboratory of South China Structural Heart Disease, Guangdong Provincial People’s Hospital, Guangdong Academy of Medical Sciences, Guangdong, China; 3 Department of Cardiology, Guangdong Cardiovascular Institute, Guangdong Provincial People’s Hospital, Guangdong Academy of Medical Sciences, Guangdong, China; 4 Shantou University Medical College, Shantou, China; 5 Guangdong Cardiovascular Institute, Guangdong Provincial Key Laboratory of South China Structural Heart Disease, Guangdong Provincial People’s Hospital, Guangdong Academy of Medical Sciences, Guangdong, China

**Keywords:** Hypertrophic obstructive cardiomyopathy, Weighted gene co-expression network analysis, Multi-factor regulatory network, Different clusters

## Abstract

**Background:**

Practical biosignatures and thorough understanding of regulatory processes of hypertrophic obstructive cardiomyopathy (HOCM) are still lacking.

**Methods:**

Firstly, public data from GSE36961 and GSE89714 datasets of Gene Expression Omnibus (GEO), Gene database of NCBI (National Center of Biotechnology Information) and Online Mendelian Inheritance in Man (OMIM) database were merged into a candidate gene set of HOCM. Secondly, weighted gene co-expression network analysis (WGCNA) for the candidate gene set was carried out to determine premier co-expressed genes. Thirdly, significant regulators were found out by virtue of a multi-factor regulatory network of long non-coding RNAs (lncRNAs), messenger RNAs (mRNAs), microRNAs (miRNAs) and transcription factors (TFs) with molecule interreactions from starBase v2.0 database and TRRUST v2 database. Ultimately, HOCM unsupervised clustering and “tsne” dimensionality reduction was employed to gain hub genes, whose classification performance was evaluated by a multinomial model of lasso logistic regression analysis binded with receiver operating characteristic (ROC) curve.

**Results:**

Two HOCM remarkably-interrelated modules were from WGCNA, followed by the recognition of 32 crucial co-expressed genes. The multi-factor regulatory network disclosed 7 primary regulatory agents, containing lncRNAs (XIST, MALAT1, and H19), TFs (SPI1 and SP1) and miRNAs (hsa-miR-29b-39 and has-miR-29a-3p). Four clusters of HOCM and 4 hub genes (COMP, FMOD, AEBP1 and SULF1) significantly expressing in preceding four subtypes were obtained, while ROC curve demonstrated satisfactory performance of clustering and 4 genes.

**Conclusions:**

Our consequences furnish valuable resource which may bring about prospective mechanistic and therapeutic anatomization in HOCM.

**Supplementary Information:**

The online version contains supplementary material available at 10.1186/s12920-021-01036-4.

## Background

Hypertrophic cardiomyopathy (HCM), defined as left ventricular hypertrophy ≥ 15 mm or ≥ 13 mm in first-degree relatives with unambiguous family history eliminating secondary causes, is the prevailing inheritable cardiomyopathy, with an estimated prevalence of 0.2% in the general population [1, 2]. Clinical manifestations of HCM range from asymptomatic to mortiferous, conducing to sudden cardiac death (SCD) in young adults to some degree [1, 3]. Noteworthily, hypertrophic obstructive cardiomyopathy (HOCM), with which approximately 25% to 70% of HCM patients are afflicted, is the hazardous category of HCM and equivalently signifies left ventricular outflow tract obstruction (LVOTO), interpreted by left ventricular outflow tract gradient (LVOTG) higher than or equivalent to 30 mmHg at rest or with load instigations [4–6]. HOCM predisposes victims to severe symptoms and encompasses diversified therapeutic modalities when compared with hypertrophic non-obstructive cardiomyopathy [7]. HCM is a hereditary myocardial disease in the majority of cases where autosomal dominant sarcomere protein mutations with regard to myofilament encoding are recognized in roughly 35% to 60% of patients and non-sarcomeric genetic mutations representing particular phenotypes such as Fabry disease (GLA gene) and FHL1-related diseases (FHL1 gene), have been identified in a considerable scale of sufferers, together with reported 25% of children [6]. Nevertheless, there has been insufficient detailed investigation into the fundamental molecular mechanisms of HCM, so has HOCM, which contributes to phenotypic heterogeneity [1].

Comprised of more than 200 nucleotides, long non-coding RNAs (lncRNAs) whose structures somewhat resemble those of messenger RNAs (mRNAs) but are not translated into proteins, participate in numerous biological processes [8, 9]. Precedent researches have established that dysregulation of lncRNAs is involved in the pathogenesis of HCM [[Bibr CR10], 11]. MicroRNAs (miRNAs), non-coding RNA molecules with about 21 nucleotides, prevent gene expression through post-transcriptional regulation. It has antecedently been observed that miRNAs such as miR-1 [12], miR-451 [13], and miR-22 [14], play an essential role in cardiac hypertrophy.

Further non-coding RNAs (ncRNAs) perform as crucial biomarkers and function as therapeutic targets in cardiovascular diseases. However, signaling pathways and regulatory networks underlying the pathogenesis of HOCM demand further elucidation, indicating an imperative need for the discovery of new indicators and regulatory targets of HOCM in future therapeutic progression.

Our research integrated public data from Gene Expression Omnibus (GEO), Gene database of NCBI (National Center of Biotechnology Information) and Online Mendelian Inheritance in Man (OMIM) database, and subsequently implemented weighted gene co-expression network analysis (WGCNA). A multi-factor regulatory network was constructed with the utilization of starBase v2.0 database as well as TRRUST v2 database and then accomplish HOCM clustering in order to explore significant regulators and genes. Our investigation intends to facilitate biological perspectives and distinguish potential biomarkers for diagnosis and treatment of HOCM.

## Materials and methods

### Materials

The datasets of GSE36961 contributed by Hebl VB et al. and GSE89714 contributed by Li Y et al. were downloaded from GEO database (https://www.ncbi.nlm.nih.gov/geo/), and the expression profiling was generated using GPL15389 (Illumina HumanHT-12 V3.0 expression beadchip) and GPL11154 (IlluminaHiSeq 2000). GSE36961 dataset contains 106 case samples and 39 normal ones and meantime, GSE89714 dataset involves 5 disease specimens and 4 healthy ones, where the case samples originate from HOCM sufferers who underwent surgical myectomy on account of LVOTO, and the control ones are donor myocardial tissues. Statistical analysis was executed using R software (version 3.6.0).

Simultaneously, genes associated with HCM were acquired from Gene database of NCBI (https://www.ncbi.nlm.nih.gov/gene/) and OMIM database (https://omim.org/).

### Data preprocessing and differential expression analysis

In GSE36961 and GSE89714 datasets respectively, probes were matched with corresponding genes with normalized expression values provided by GEO database, following the removal of invalid probes, in which median value was selected as expression level of the gene when multiple probes were applied to the same gene. Afterwards, differentially expressed genes (DEGs) were calculated from “limma” algorithm in two respective datasets. |log_2_FC| (fold change) > 0.58 and adjusted P-value (adj. *P*. Val.) < 0.05 were considered statistically significant [15, 16].

### Identification of candidate gene set and function analysis

The DEGs from the above two datasets, in company with the HCM-associated genes from GENE and OMIM databases were merged, whose redundancy was removed to establish candidate gene set. The expression profile data of candidate gene set in GSE36961 were extracted for the supervenient execution of WGCNA.

The “ClusterProfiler” software package from R language was employed to implement enrichment analysis of candidate gene set including Kyoto Encyclopedia of Genes and Genomes (KEGG) and Gene Ontology (GO) enrichment analysis.

### WGCNA

WGCNA is a systematic biological approach to build up a scale-free network through gene expression data. Initially, a gene co-expression similarity matrix is constructed by computing Pearson correlation coefficients between pairs of genes. Secondly, the similarity matrix is converted into a weighted adjacency matrix via introducing an acceptable soft-thresholding (β) meaning β power Pearson correlation coefficient to generate a scale-free network. What is more, topological overlap measure (TOM), describing relationship within genes, leads to the adjacency matrix transforming into topological matrix, while 1-TOM suggests dissimilarity of genes. Fourthly, TOM-based dissimilarity promotes average linkage hierarchical clustering of genes, and module identifying will be achieved relying on dynamic shear tree. Lastly, trait-correlated modules will be acquired, figuring module eigengenes (ME) referring to the most representative gene in modules, module membership (MM) implying membership of genes in respective modules, and gene significance (GS) measuring relation degree between genes and external information, where GS and MM show a high correlation [17].

Our experiment operated the “WGCNA” package of R software to constitute the weighted co-expression network for the candidate gene set. After screening HOCM strongly-correlated modules, the “ClusterProfiler” software package was engaged for function enrichment analysis of genes from those modules. Based on biological process (BP) and pathway enrichment analysis, significant GO terms and pathways were identified (adj. *P*. Val. < 0.05). Meanwhile, co-expressed gene sets within strongly-correlated modules were constructed into an interaction subnetwork, with a succeeding detection of core genes of modules according to network node degrees by means of Cytoscape3.7.2 program to visualize the network [18, 19].

### Construction of the multi-factor regulatory network

Aiming to uncover miRNAs, lncRNAs and transcription factors (TFs) regulating the core genes, interaction pairs between ncRNAs and their target genes from starBase v2.0 (http://starbase.sysu.edu.cn/) database involving miRNA-lncRNA and miRNA-mRNA couples, and interaction ones between TFs and corresponding target genes (TF-mRNA) from TRRUST v2 database (https://www.grnpedia.org/trrust/) were downloaded [20, 21].

MiRNA-lncRNA and miRNA-mRNA pairs which conform to number of supporting experiments greater or equal to high stringency (≥ 3), would be chosen for further analysis. In addition, miRNA-mRNA matches should be identified by at least one of the following softwares that consisted of targetScan, picTar, RNA22, PITA and miHCMnda/mirSVR.

MiRNAs and TFs connected with the above crucial genes would be filtrated, accompanied by the recognition of lncRNAs interacted with the preceding miRNAs. Interplay pairs of core genes containing miRNA-lncRNA, miRNA-mRNA and TF-mRNA, were organized to create a multi-factor regulatory network. By calculating the degree of each regulator (miRNA, lncRNA, and TF), the regulators with degree > 5 were regarded as critical.

For the purpose of detecting functions and signal channels of critical regulators, key genes interrelated with the regulators from former miRNA-lncRNA, miRNA-mRNA and TF-mRNA couples would be figured out to execute enrichment analysis.

### Clusters of HOCM and identification of hub genes

The essential co-expressed genes derived from WGCNA modules were mapped to GSE36961 to apply K-means unsupervised clustering method combined with “tsne” dimensionality reduction to divide all HOCM samples (N = 106) into several clusters. The optimal K value (number of categories) was determined on the basis of searching the ideal inflection point of SSE (sum of the squared error, i.e. quadratic sum of distances of all points to the center of clusters to which they belong or quadratic sum of error). After that, expression patterns of these vital genes in different subtypes were examined and genes with significant difference in distinct clusters were analyzed, which might act as promising marker genes in HOCM clusters. A multinomial model of lasso logistic regression analysis would be established with the “glmnet” package in R, adopting expression profiles of hub genes as independent variables and clusters of HOCM as dependent variables. Whereafter, receiver operating characteristic (ROC) curve of the model and genes would be plotted, where area under the curve (AUC) of ROC curve evaluated their performance [22].

## Results

### Data preprocessing and differential expression analysis

**Fig. 1:**
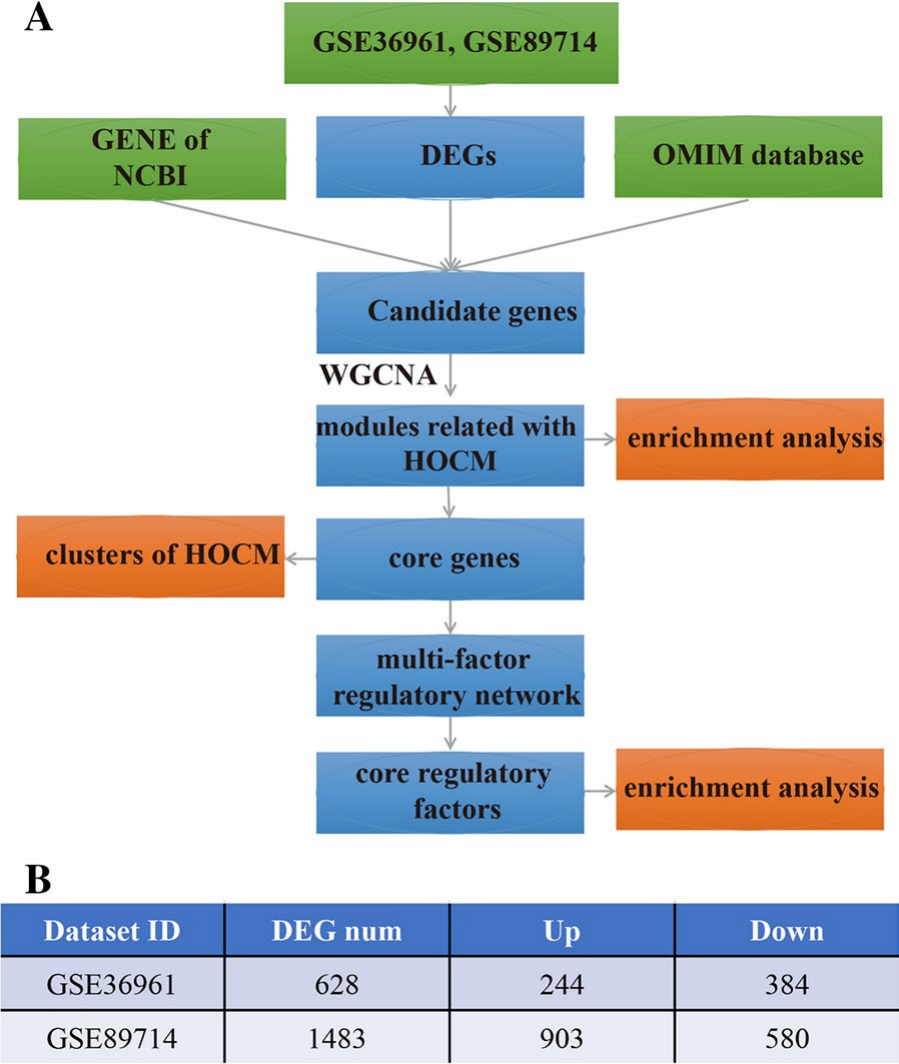
Data preprocessing and differential expression analysis **a** workflow used for bioinformatics analyses; **b** DEGs in two datasets

The steps conducted in our study were exhibited in Fig. 1a. Probes with zero expression inside 80% of specimens were excluded in GSE110226 and GSE89714 datasets respectively, and remaining probes matched with genes were embraced for differential expression analysis. DEGs of the two datasets were presented in Additional file 1: Table S1, where a total of 628 DEGs were identified from GSE36961 (|log_2_FC|> 0.58, adj. *P*. Val. < 0.05) between HOCM samples and normal tissues, which included 244 upregulated and 384 downregulated genes, and besides, 1483 DEGs were extracted from GSE89714 (|log_2_FC|> 0.58, adj. *P*. Val. < 0.05), encompassing 903 upregulated and 580 downregulated genes (Fig. 1b). The volcano plots (Additional file 2: Fig. S1) displayed the DEGs.

### Identification of candidate gene set and function analysis

**Fig. 2:**
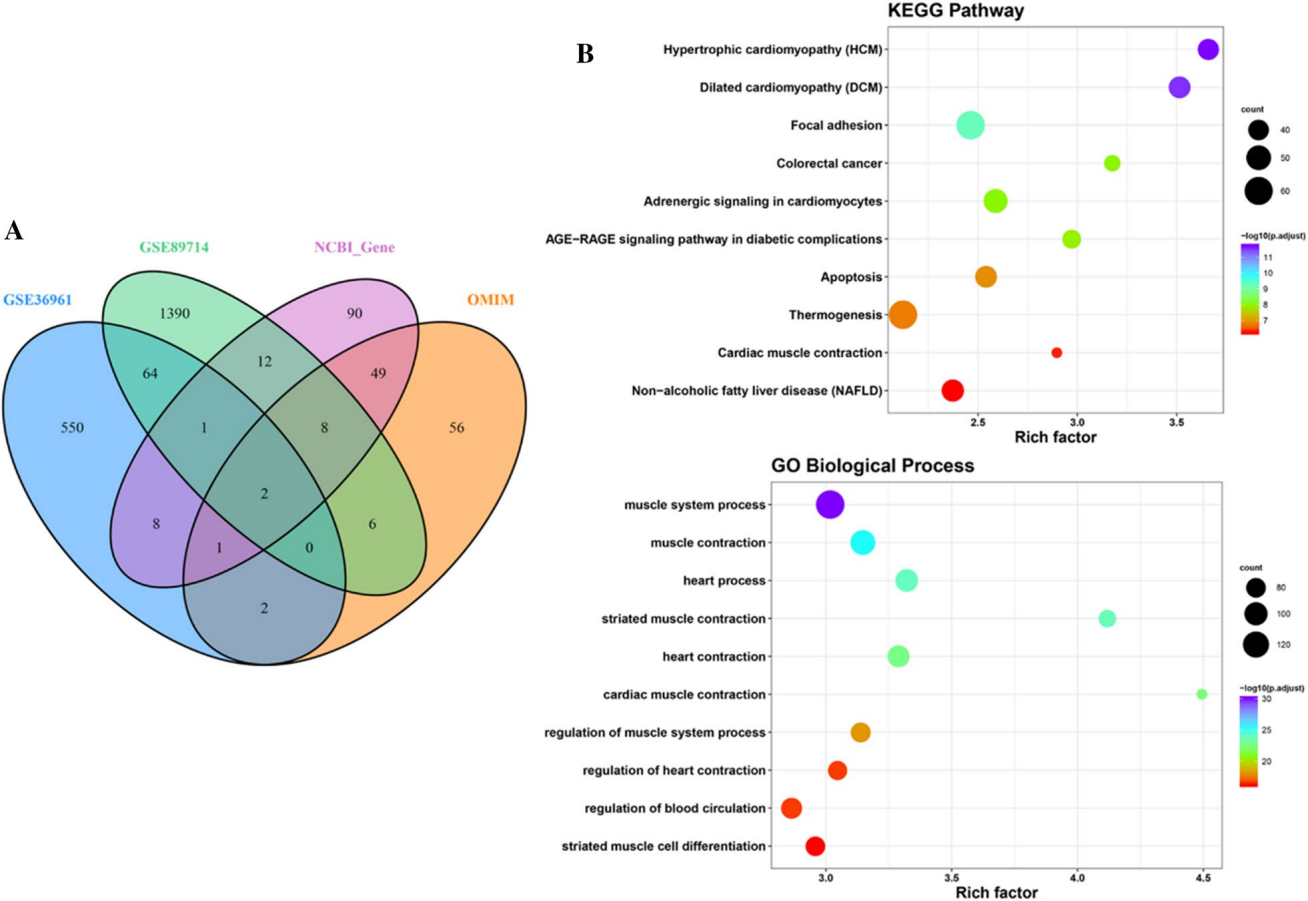
Identification of candidate gene set and function analysis **a** identification of candidate gene set; **b** KEGG pathway and BP analyses of candidate gene set, KEGG: Kyoto Encyclopedia of Genes and Genomes; BP: biological process

Totally, 171 HCM-related genes were attained from Gene database of NCBI and 124 genes from OMIM database (Additional file 3: Table S2). These HCM-associated genes were combined with the DEGs of two datasets and the redundancy were removed to get 2239 candidate genes, considering as a candidate gene set (Fig. 2a). Furthermore, KEGG function enrichment analysis of candidate genes suggested that they showed significant enrichment in HCM and dilated cardiomyopathy pathways, and BP of GO analysis involved in muscle system process and heart process (Fig. 2b and Additional file 4: Table S3).

### WGCNA

**Fig. 3:**
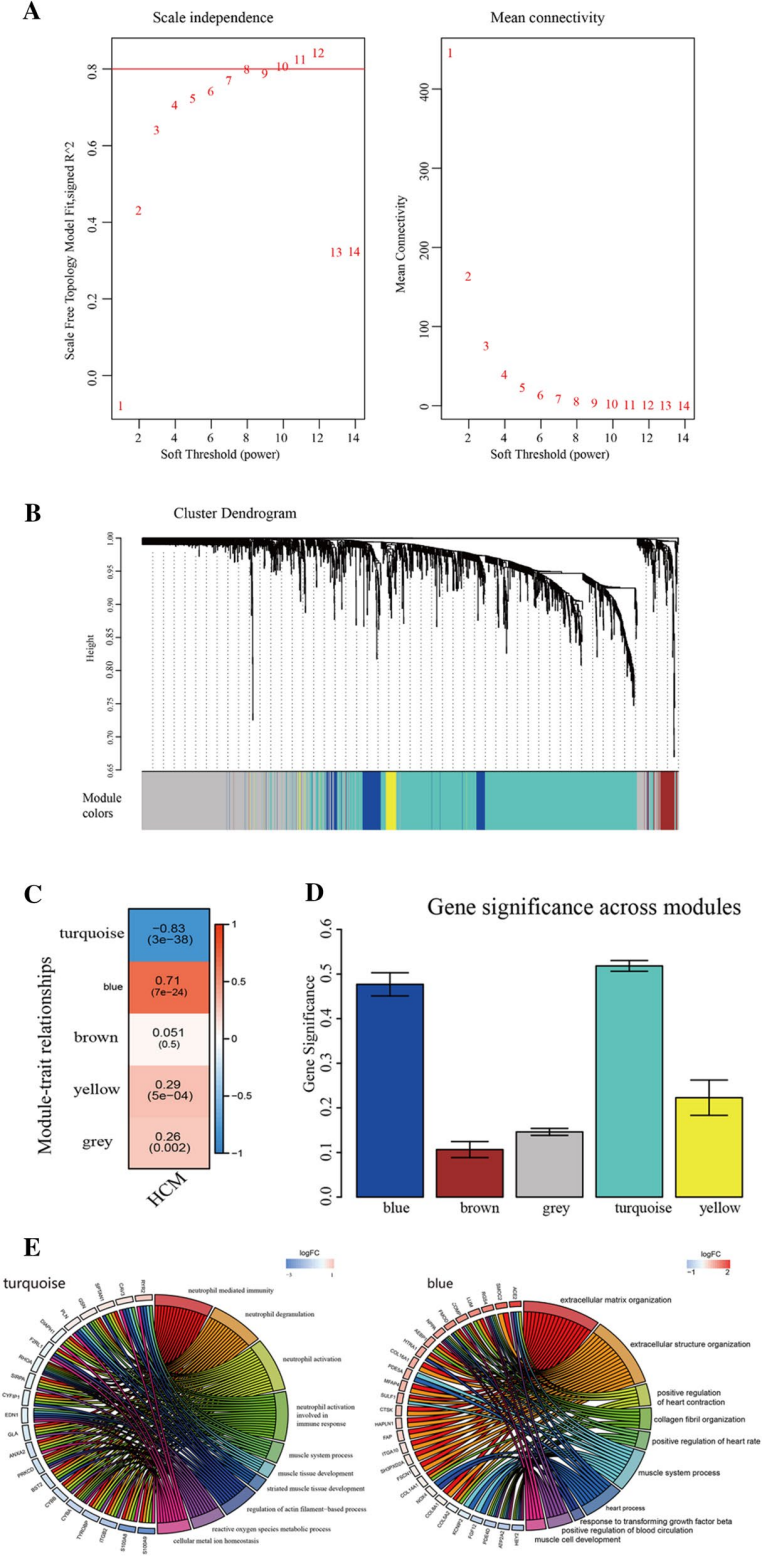
Overview of WGCNA network construction of the candidate gene set **a** identification of soft threthold; **b** the candidate gene set divided into 5 modules; **c** the module−trait relationships of HOCM in 5 modules; **d** Gene significance (GS) in the modules, and the larger the GS score is, the larger the difference is; **e** BP function analysis of the two modules, and the larger the logFC (fold change) score is, the larger the difference is

1977 genes of gene expression profile data which expressed in GSE36961 based on 2239 candidate genes, were applied to form WGCNA network (Additional file 5: Table S4). The results of clustering revealed no outlier sample, leading to subsequent analysis for all 145 samples in GSE36961. In a scale-free co-expressed network, there is a negative correlation between logarithm of nodes with k connectivity [log(k)] and logarithm of probability of those nodes presence [log(P(k))], with the correlation coefficient > 0.8 required. A soft threshold (β) value of 8 was set to produce such a network and appropriate network connectivity (Fig. 3a). Depending on TOM-based average-linkage hierarchical clustering method and dynamic shear tree, once setting a minimum number of genes for each gene network module to be 30, along with determining gene modules by dynamic shear method, eigengenes of each module were computed at one time, and module clustering analysis came behind. A height of 0.25 was configured, which means dissimilarity of genes and is consistent with similarity threshold being 0.75, and modules with relatively close distance were merged into new modules. To sum up, 5 modules were attained (Fig. 3b and Additional file 6: Table S5).

Pearson correlation coefficients of expression profiles in every module between ME and genes in HOCM specimens were calculated. The bigger absolute value of Pearson correlation coefficient is, the more important module is supposed as in HOCM. Subsequently, GS value of every module was counted, where a higher value of GS represented the module is more relative with HOCM. Finally, the turquoise module (cor = 0.82, *p* < 1e−200) and blue module (cor = 0.72, *p* < 4.3e−25) were thought as the most relative modules with HOCM (Fig. 3c, d and Additional file 2: Fig. S2). The BP of GO enrichment analysis of genes from the two modules revealed that genes in the turquoise module engaged into muscle system process and muscle tissue development, and genes from the blue module participated with muscle system process and heart process (Fig. 3e and Additional file 6: Table S5).

### Construction of the multi-factor regulatory network

**Fig. 4:**
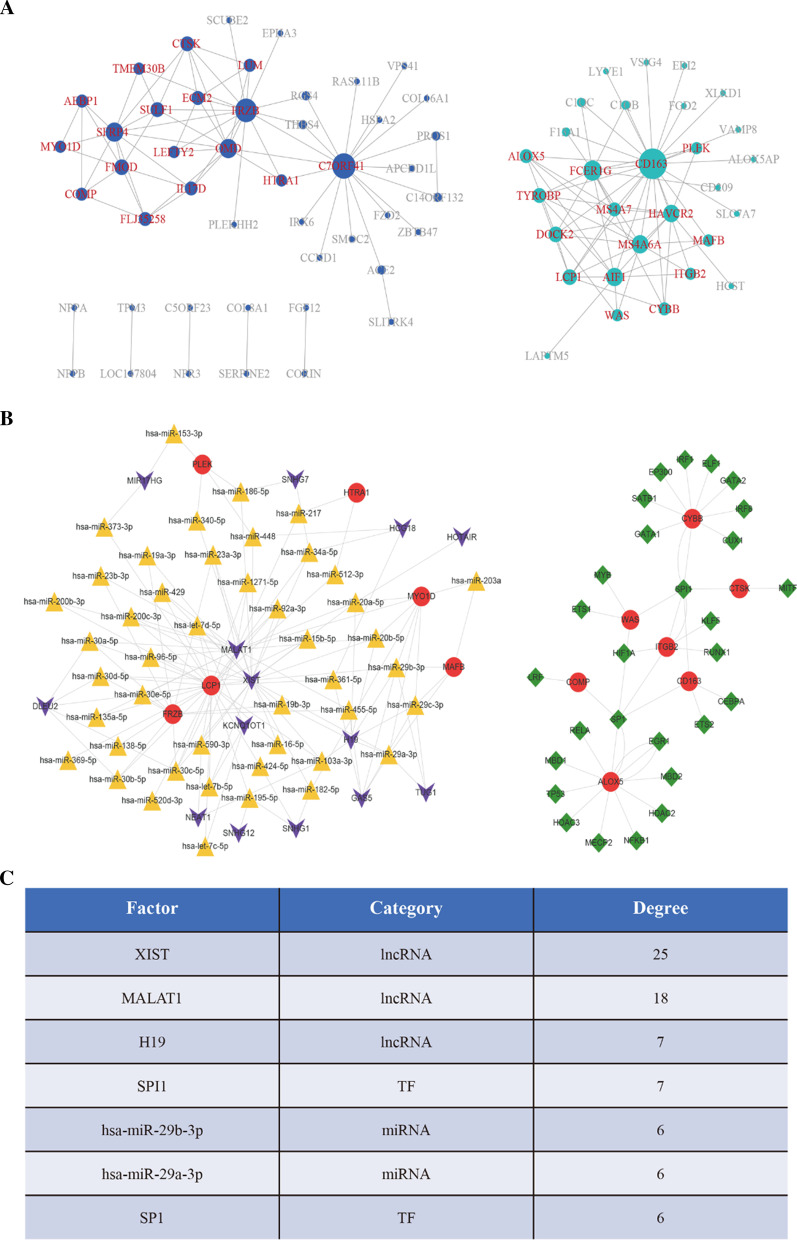
Construction of multi-factor regulatory network **a** Core genes in the blue module were 17 (left), and core genes in the turquoise module were 15 (right), where core genes were labeled in the red color. **b** The construction of multi-factor regulatory network was done by Cytoscape software, where the green rhombuses represent transcription factors (TFs), the red circles represent genes (mRNAs), the orange triangles represent miRNAs, and the purple arrows represent lncRNAs. **c** Seven key regulatory factors were found in the network, where the higher the degree is, the more important the regulatory function in the network is

Genes from the two modules were anatomized with the intention of identifying hub genes interrelated with HOCM. In accordance with gene expression relationship in the turquoise module, co-expression pairs with a connection threshold value of no less than 0.2 were chosen as edges of co-expression network to construct a turquoise module network diagram, and genes with a node degree greater or equal to 3 (N = 15) were picked out as core genes of the turquoise module. Similarly, a connection threshold value of no less than 0.07 was fixed and genes with a node degree of greater or equal to 3 (N = 17) were found out as key genes of the blue module. These 32 core genes were served as vital co-expression genes for comprehensive anatomization (Fig. 4a and Additional file 7: Table S6).

376 miRNA-lncRNA interactions and 160,774 miRNA-mRNA interactions were completely caught from starBase v2.0 database, and human 9396 TF-mRNA interaction pairs were gathered from TRRUST v2 database (Additional file 8: Table S7). MiRNAs and TFs that interact with the co-expressed key genes (N = 32) were screened, followed by selecting lncRNAs that communicate with these miRNAs, and a multi-factor regulatory network with 175 interaction pairs was finally generated (Fig. 4b and Additional file 9: Table S8).

Employing the Mcode plugin of Cytoscape to calculate degrees of every regulatory factor (miRNA, lncRNA, and TF), the first 7 regulatory factors (degree > 5) were distinguished as the predominant ones, comprising 3 lncRNAs (XIST, MALAT1, and H19), 2 TFs (SPI1 and SP1) and 2 miRNAs (hsa-miR-29b-3p and has-miR-29a-3p) (Fig. 4c and Additional file 9: Table S8).

8397 target genes that interlinked with 7 predominant regulators from miRNA-lncRNA, miRNA-mRNA and TF-mRNA interaction pairs were selected. Enrichment analysis for these target genes presented that they are connected to biological processes such as positive regulation of catabolic process, histone modification and proteasomal protein catabolic process (Additional file 2: Fig. S3 and Additional file 9: Table S8).

### Clusters of HOCM and identification of hub genes

**Fig. 5:**
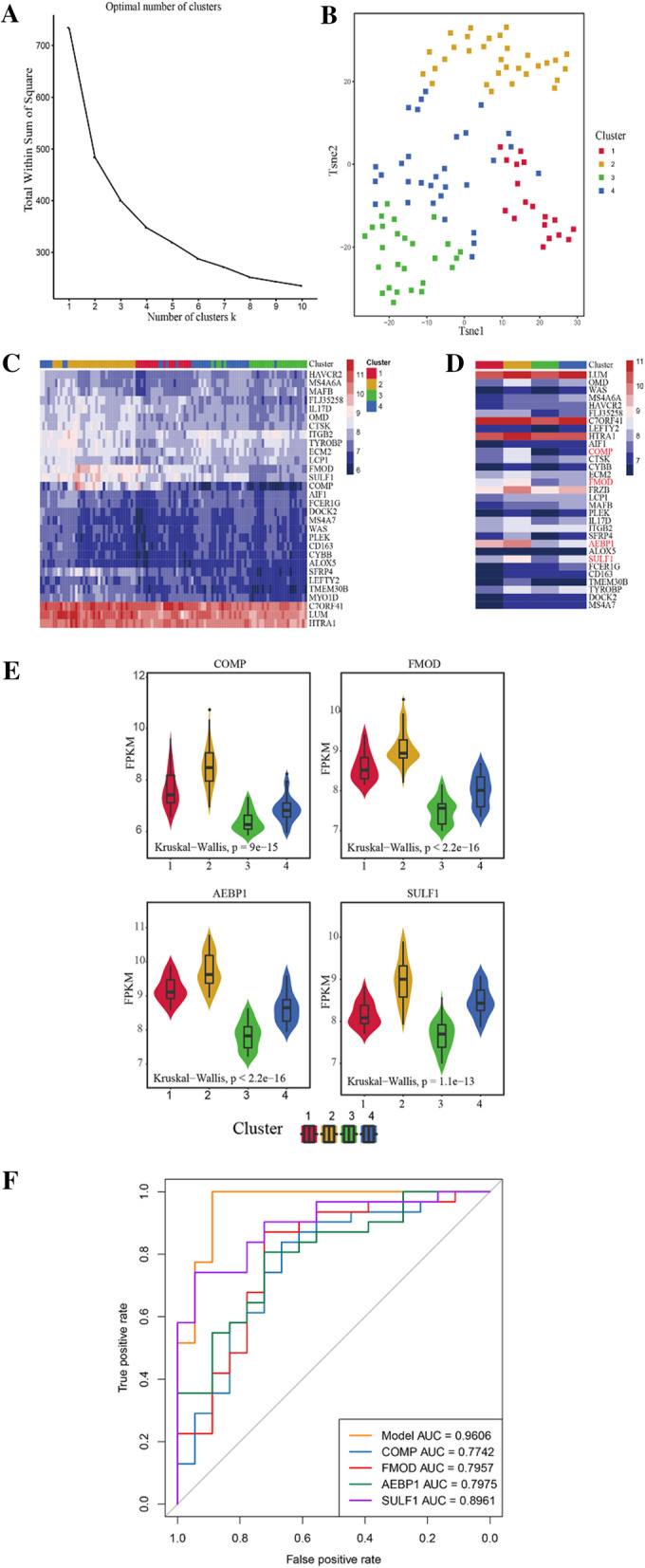
Clusters of HOCM and identification of hub genes **a** K = 4 was selected as the optimal number of clusters since the K value is decreased by a negligible amount. **b** The tSNE algorithm provided each sample with a unique x- and y-coordinate (tSNE1 and tSNE2) according to each sample’s gene expression of 32 core genes. All HOCM samples were clearly divided into 4 clusters. **c** The expression of core genes in all HOCMs was shown in the heatmap. **d** The expression of core genes in 4 clusters was shown in the heatmap. **e** COMP, FMOD, AEBP1 and SULF1 showed significant expression in different clusters. **f** Receiver operating characteristic (ROC) curves of the model and 4 hub genes validated the classification performance of 4 genes

The 32 co-expressed principal genes were mapped to GSE36961 for K-means unsupervised clustering, and an optimal K value of 4 was settled since SSE presented a slow tendency of decline after K = 4 (Fig. 5a and Additional file 10: Table S9). The “tsne” R software package was held to administer dimensionality reduction for gene expression data, where all HOCM samples were also evidently divided into 4 clusters. Expression of 32 genes in the whole HOCM collections was shown in the heatmap, emerging high uniformity with previous clustering (Fig. 5b, c and Additional file 10: Table S9). It was rationally speculated that these 32 genes were of great significance for HOCM clustering.

Furthermore, difference of 32 co-expressed hub genes in different clusters was investigated, and the average value of each gene in each class was taken as the gene expression value in that class. What stands out in the procedure is that 4 genes including COMP, FMOD, AEBP1 and SULF1 manifested significant expression (*p* < 0.01) in different groups (Fig. 5d). The four genes were extracted for expression distribution in all HOCM collections with the aim of exploring how they expressed in distinct classifications. It was apparent that there was a significant difference (*p* < 0.01) of their expression in four clusters (Fig. 5e). The AUCs of the model and 4 genes (COMP, FMOD, AEBP1 and SULF1) are 0.9606, 0.7742, 0.7957, 0.7975 and 0.8961, respectively (Fig. 5f).

## Discussion

HOCM is almost inherited with sarcomeric and non-sarcomeric causes participated, and has been one notable risk factor of SCD in young individuals [6]. However, few previous studies dig out underneath precise molecular indicators and regulatory mechanisms. Our project was undertaken to scrutinize potential biosignatures and fuel further study endeavours to uncover underlying pathophysiological mechanisms of HOCM with high heterogeneity.

In our entire study, two datasets were integrated from GEO database on HOCM with associated genes of HCM in GENE and OMIM databases to obtain the candidate gene set. Then, WGCNA method was used to identify related modules of HOCM. The integration of high-throughput data, online databases and bioinformatic method for scale-free network have widened the disease spectrum and strengthened the evidence. BP analysis indicated that the candidate gene set and genes in most of the relevant modules were concentrated on muscle system process, muscle contraction and heart process. Pathway analysis demonstrated that the candidate gene set was mostly enriched in HCM, focal adhesion and dilated cardiomyopathy. These results exhibited correlation with HOCM, and consequently, 32 co-expressed genes with the highest degree in two highly-connected modules were designated as core genes.

The miRNAs, lncRNAs and TFs that interact with the co-expressed key genes were then screened to obtain a multi-factor regulatory network. To date, several studies have reported features of ncRNAs in HOCM [23, 24]. Nevertheless, details of RNA crosstalk in HOCM have not been thoroughly elucidated. In this exploration, a comprehensive lncRNA-miRNA-mRNA-TF regulatory network was founded, expounding views on gene regulation at pre-transcriptional and post-transcriptional levels. Moreover, bioinformatics technology was applied to explore vitally important molecules that are involved in the development of HOCM, which might be served as felicitous candidate markers for future therapy. The primary 7 regulatory factors were found as the ones of essential significance, which covered lncRNAs (XIST, MALAT1, and H19), TFs (SPI1 and SP1) and miRNAs (hsa-miR-29b-39 and has-miR-29a-3p). XIST, called lncRNA X-inactive specific transcript, has been described as a necessary regulator of cardiac hypertrophy by modulating miR-101 [25] and miR-330 [26]. XIST also exposed certain association with heart failure in females [27]. In vivo experiments unveiled that knockdown of XIST can inhibit myocardial cell apoptosis in acute myocardial infarction rat model by adjusting miR-449 [28]. Besides, H19 has been identified as a regulator that targets PPARα of cardiac hypertrophy [29, 30]. The results disclosed that XIST, MALAT1 and H19 possibly regulated other miRNAs involved in cardiac hypertrophy, such as miR-15b [31], miR-19b [32] and miR-29b [33] in our research.

The miRNAs and TFs consistent with other researches were identified. MiR-29 is a regulatory agent of cardiomyocyte hypertrophy via Wnt and mTOR signaling pathways [34, 35]. Moreover, SP1 is capable of influencing cardiomyocyte hypertrophy by inducing lncRNA CTBP-AS2 [[Bibr CR36]] and SP1/GATA4 signaling pathways [37]. Interestingly, SPI1 has not been reported in cardiac hypertrophy so far.

HOCM clustering unveiled consistent classification effect with "tsne" dimensionality reduction, which may be interpreted by heterogeneity of HOCM. The classification performance of 4 genes (COMP, FMOD, AEBP1 and SULF1) expressing with significant difference in four clusters was verified by the multinomial model of lasso logistic regression analysis and ROC curve. It's worth noting that there is no relevant report about the roles of COMP, FMOD, AEBP1 and SULF1 in HOCM or even myocardial hypertrophy, implying the need for further exploration. Although no classifying difference was observed from the expression of a single gene, the HOCM samples were separated into 4 clusters obviously by combining the 32 co-expressed key genes. From this point, we speculated that these 32 co-expressed key genes are of great significance for HOCM typing and the 4 genes are considered as important biomarkers due to different progressive stages or prognosis of HOCM.

Nonetheless, the generalisability of these results is subject to certain limitations. Firstly, our enquiry emphasized data mining and analyzation without experimentalconfirmation. Secondly, due to lack of relevant prognostic information, clinical classification of HOCM and survival analysis associated with key genes were not conducted.

## Conclusions

The most obvious finding to emerge from our current study is to seek out 32 premier genes and 7 regulatory factors that might be developed into biological indicators in HOCM. Categorization of HOCM samples acquired from co-expression of hub genes demonstrated a satisfactory classifying effect. Four genes manifested significant difference in different subclasses and could be converted into novel biosignatures for varying hypertrophic cardiomyopathy subtypes. The present study lays the groundwork for prospective research into detecting promising biomarkers, therapeutic targets and prognostic indicators to enhance competences to diagnose, counsel and treat HOCM patients.

## Supplementary Information

**Additional file 1: Table S1.** The list of DEGs (top 50) in GSE36961 and GSE89714 datasets.

**Additional file 2. Fig. S1.** Volcano plots showing differentially expressed genes (DEGs). **Fig. S2.** The scatter plot of gene significance (GS) versus module membership (MM) for the turquoise module and the blue module. **Fig. S3.** The BP analysis of targeted genes regulated by core regulators.

**Additional file 3: Table S2.** HCM-related genes from OMIM and NCBI database.

**Additional file 4: Table S3.** The BP and KEGG analysis (top 10) of candidate genes.

**Additional file 5: Table S4.** Candidate genes and gene expression values for WGCNA. The details in supplementary materials.

**Additional file 6: Table S5.** The BP analysis of genes in the turquiose module and the blue module.

**Additional file 7: Table S6.** The turquoise and blue modules network analysis.

**Additional file 8: Table S7.** The miRNA-lncRNA, miRNA-mRNA and TF-mRNA pairs from starBase v2.0 database and TRRUST v2 database.

**Additional file 9: Table S8.** Multi-factor regulatroy network with 175 interaction pairs and BP terms of target genes of core regulators.

**Additional file 10: Table S9.** Cluster of samples and core genes expression values in four clusters.

## Data Availability

Public data are deposited at Gene Expression Omnibus (GEO, https://www.ncbi.nlm.nih.gov/geo/, GSE36961 and GPL15389, GSE89714 and GPL11154), Gene database of NCBI (National Center of Biotechnology Information, https://www.ncbi.nlm.nih.gov/gene/), Online Mendelian Inheritance in Man (OMIM, https://omim.org/) databases. All data analyzed in this study are included in the manuscript and supplementary materials.

## Abbreviations

HOCM: hypertrophic obstructive cardiomyopathy
HCM: hypertrophic cardiomyopathy
GEO: Gene Expression Omnibus
NCBI: National Center of Biotechnology Information
OMIM: Online Mendelian Inheritance in Man
WGCNA: weighted gene co-expression network analysis
ROC: receiver operating characteristic
SCD: sudden cardiac death
LVOTO: left ventricular outflow tract obstruction
LVOTG: left ventricular outflow tract gradient
TF: transcription factor
DEG: differentially expressed gene
KEGG: Kyoto Encyclopedia of Genes and Genomes
GO: Gene Ontology
BP: biological process
TOM: topological overlap measure
ME: module eigengene
MM: module membership
GS: gene significance
SSE: sum of the squared error
