# Supplementary material for: Multi-factor regulatory network and different clusters in hypertrophic obstructive cardiomyopathy
Source: BMC Med Genomics. 2021 Aug 6;14:199. doi: 10.1186/s12920-021-01036-4 (PMC8348869; doi:10.1186/s12920-021-01036-4)

**Additional file 2**

**Supplementary Figure 1:** Volcano plots showing differentially expressed genes (DEGs).


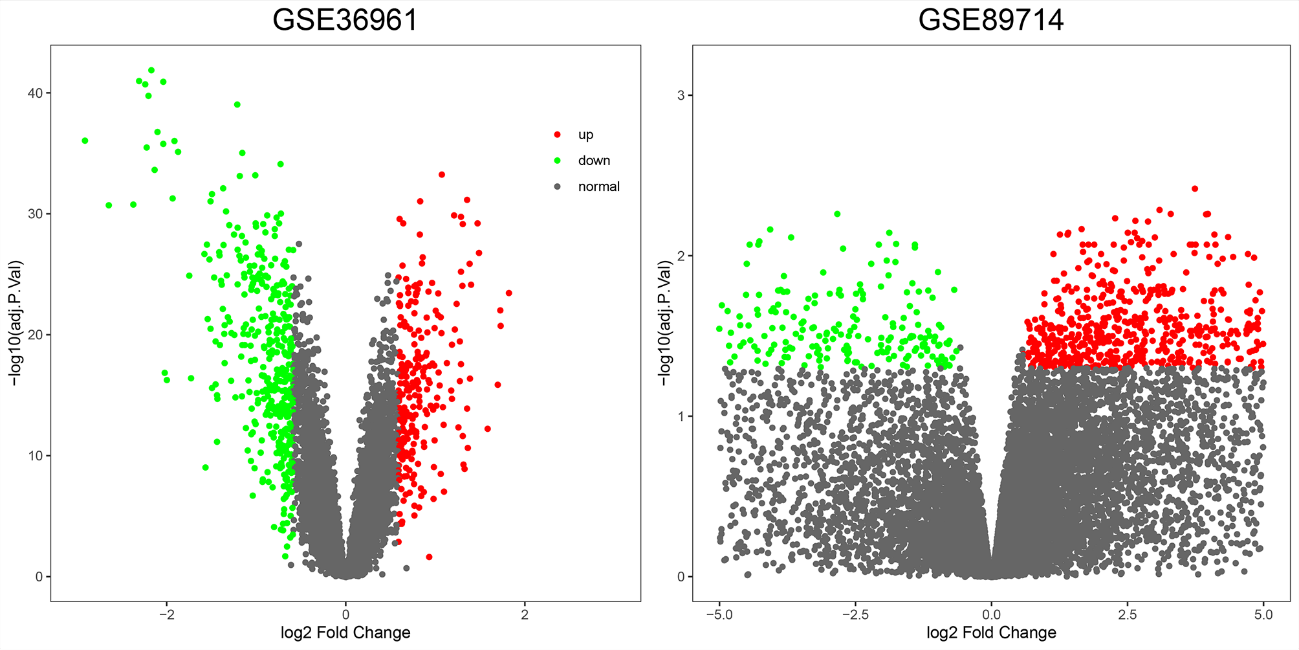


**Supplementary Figure 2:** The scatter plot of gene significance (GS) versus module membership (MM) for the turquoise module and the blue module.


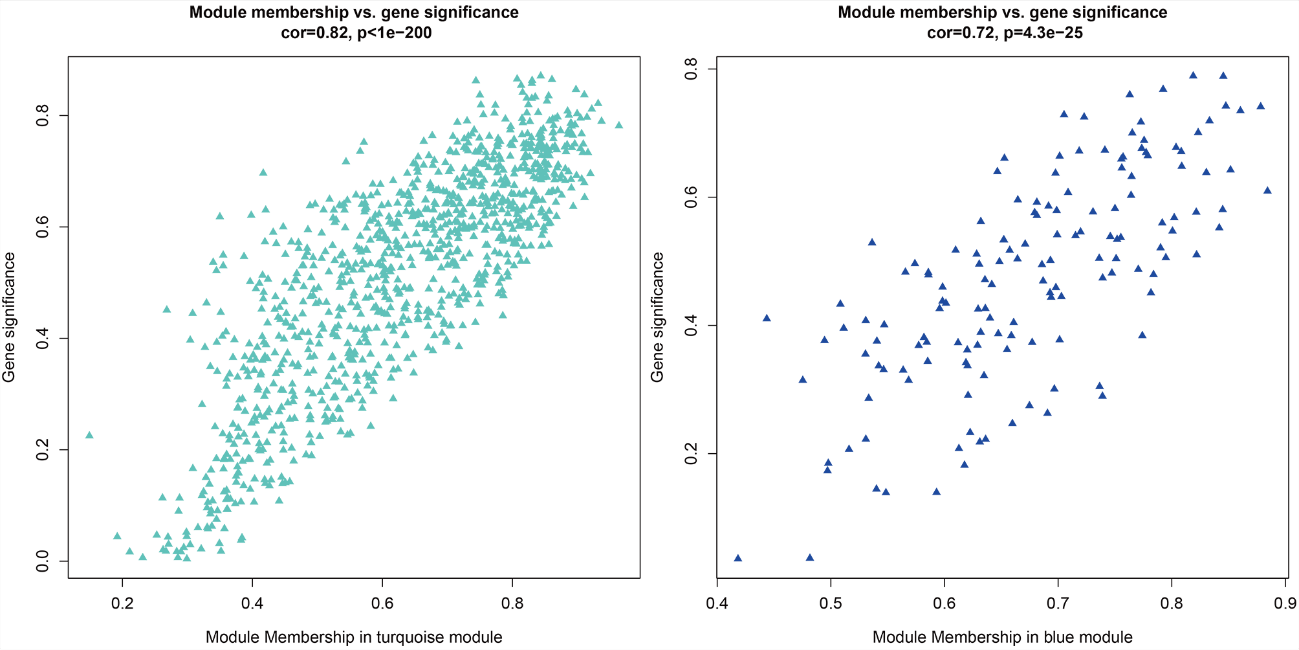


**Supplementary Figure 3:** The BP analysis of targeted genes regulated by core regulators.


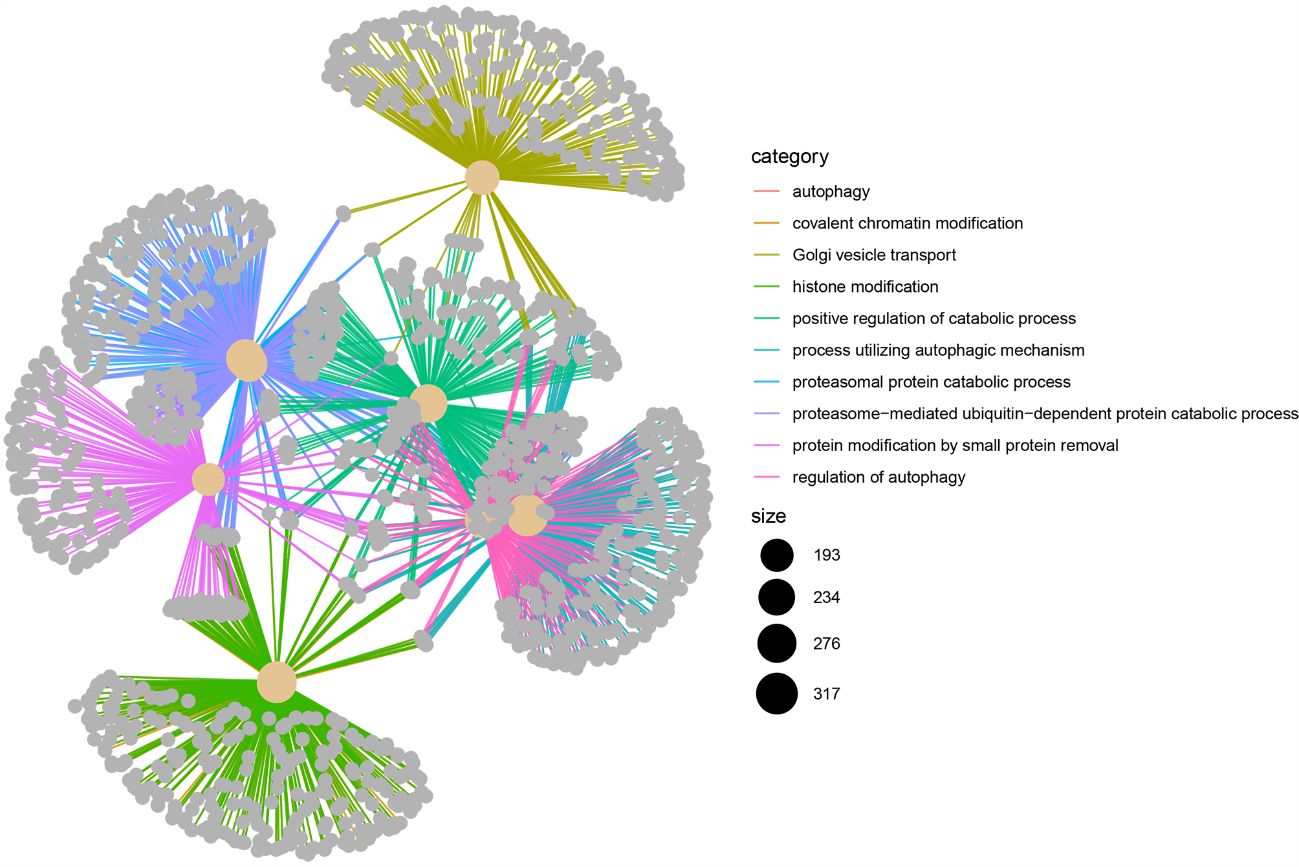

Supplement: Supplementary file 2 — Additional file 2. Fig. S1. Volcano plots showing differentially expressed genes (DEGs). Fig. S2. The scatter plot of gene significance (GS) versus module membership (MM) for the turquoise module and the blue module. Fig. S3. The BP analysis of targeted genes regulated by core regulators. [file 12920_2021_1036_MOESM2_ESM.docx]
